# Silent but Not Static: Accelerated Base-Pair Substitution in Silenced Chromatin of Budding Yeasts

**DOI:** 10.1371/journal.pgen.1000247

**Published:** 2008-11-07

**Authors:** Leonid Teytelman, Michael B. Eisen, Jasper Rine

**Affiliations:** 1 Department of Molecular & Cell Biology, University of California Berkeley, Berkeley, California, United States of America; 2 California Institute for Quantitative Biosciences, Berkeley, California, United States of America; 3 Center for Integrative Genomics, University of California Berkeley, Berkeley, California, United States of America; Stanford University School of Medicine, United States of America

## Abstract

Subtelomeric DNA in budding yeasts, like metazoan heterochromatin, is gene poor, repetitive, transiently silenced, and highly dynamic. The rapid evolution of subtelomeric regions is commonly thought to arise from transposon activity and increased recombination between repetitive elements. However, we found evidence of an additional factor in this diversification. We observed a surprising level of nucleotide divergence in transcriptionally silenced regions in inter-species comparisons of *Saccharomyces* yeasts. Likewise, intra-species analysis of polymorphisms also revealed increased SNP frequencies in both intergenic and synonymous coding positions of silenced DNA. This analysis suggested that silenced DNA in *Saccharomyces cerevisiae* and closely related species had increased single base-pair substitution that was likely due to the effects of the silencing machinery on DNA replication or repair.

## Author Summary

Many plants, fungi, pathogens, and animals have chromosome regions that are silenced. Special proteins change the chromosome structure in these domains, turning genes off or lowering their expression levels. We found an increased frequency of DNA mutations in these silenced regions of closely related yeasts. This increase is likely due to silencing proteins interfering with DNA repair or replication. Accurate replication of genetic information with minimal mutations is usually critical for the survival and fitness of an organism; however, there are examples where a high mutation rate is beneficial. The silenced regions of chromosomes are often associated with virus-like transposable elements, and with genes that are important in responding to environmental changes. Hence, it is possible that elevated DNA mutations in silenced regions contribute to genome defense against transposable elements or increased genetic diversity to cope with variation in surrounding conditions.

## Introduction

The ends of chromosomes in yeasts, vertebrates, *Drosophila*, and eukaryotic pathogens such as *Plasmodim falciparum* diverge more rapidly than the rest of their genomes [1]. In budding yeasts of the genus *Saccharomyces*, chromosome ends contain a high density of repeated sequences and relatively few genes; they are more diverged between species than any other portions of the genomes, and are highly variable within species [2],[3]. The accelerated diversification of subtelomeric DNA is commonly attributed to the presence of transposons and the repetitive nature of these regions, as both contribute to recombination between different chromosome ends [4],[5]. However, subtelomeric regions in yeasts are also silenced, analogously to metozoan heterochromatin [6], raising the possibility that the formation and maintenance of a silenced chromatin state contribute to the observed rapid evolution.

**Figure 1:**
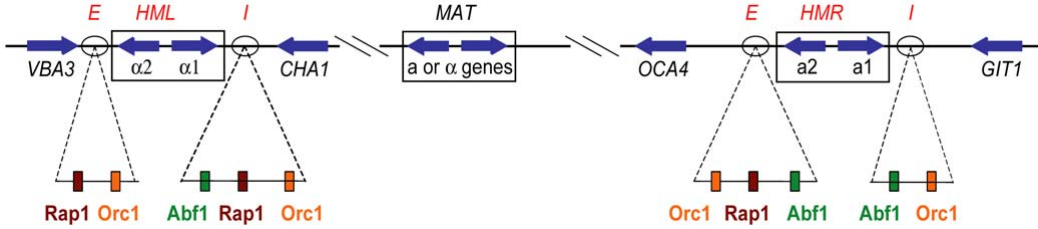
Chromosome III mating loci. *MAT* and the cryptic mating loci on chromosome III of *S. cerevisiae*. The genes in the mating loci, *HML*- and *HMR*-neighboring genes, the *E* and *I* silencers, and the binding sites for ORC, Rap1, and Abf1 in the silencers are shown. The boxes around the mating-type genes represent the sequences shared between the *MAT* and the *HML* and *HMR* loci. The *Saccharomyces cerevisiae* genome feature coordinates are in Table S2.

In *S. cerevisiae*, the best characterized silenced regions are the *HML* and *HMR* transcriptionally inactive mating loci of chromosome III. They contain non-expressed copies of the *MAT* ***a*** and *MAT* ***α*** mating-type genes. During mating type interconversion, *HML* or *HMR* is copied into the *MAT* locus, also on chromosome III, where the resident allele is transcribed. Since haploid cells that express both *MAT* **a** and *MAT* **α** behave as non-mating diploids, it is crucial that *HML* and *HMR* are silenced. This is achieved through the *E* and *I* silencers that flank both of the silenced loci (Figure 1) and recruit Silent Information Regulator (Sir) proteins which then spread throughout the regions. The Sir proteins bind to and deacetylate the tails of histones H3 and H4, leading to silencing of *HML* and *HMR* [7].

The Sir2/Sir3/Sir4 protein complex that is responsible for *HML* and *HMR* silencing also binds to subtelomeric regions of *S. cerevisiae* chromosomes [8]. In contrast to the strong and robust silencing of *HML* and *HMR*, subtelomeric silencing is weaker [9]. Nevertheless, native telomere-proximal genes and reporter genes inserted near telomeres are reliably silenced [10]–[13].

The *Saccharomyces sensu stricto* species (*S. paradoxus*, *S. mikatae*, *S. kudriavzevii*, *S. bayanus*) genome sequences are sufficiently closely related to allow identification of conserved regulatory sequences [14]. Essentially all *S. cerevisiae* protein-coding genes are found in these other species, and most orthologous intergenic regions in the *sensu stricto* yeasts can be readily aligned [2],[15]. However, in analyzing the evolution of the *HML* and *HMR* silencers, we discovered a surprising lack of DNA conservation in all four flanking regions, motivating an in-depth exploration of the evolution of silenced regions within and between these yeast species. Our observations suggested an additional force in the shaping of these regions.

## Results

### Lack of Cross-Species Conservation in Sequences Flanking *HML* and *HMR*

**Figure 2:**
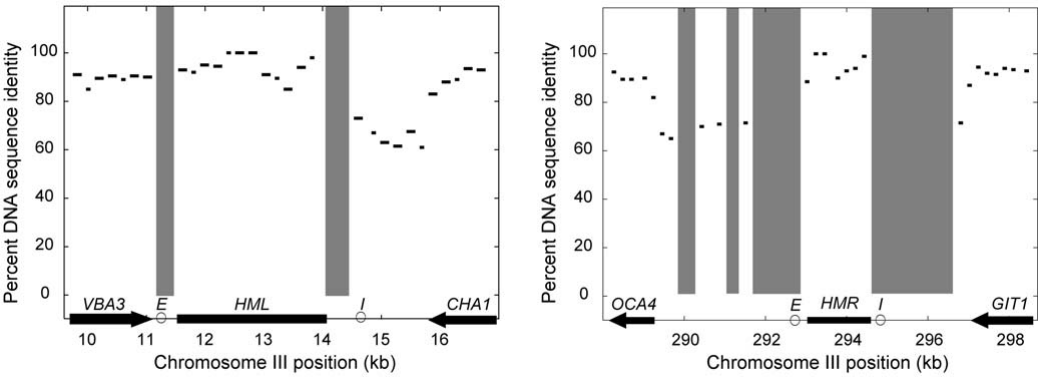
Lack of conservation in *HML* and *HMR* flanking intergenic regions. The results from the BLAST searches with *S. cerevisiae HML* and *HMR* and surrounding sequence against corresponding syntenic *S. paradoxus* contigs are shown with percent identity plotted for 200-bp windows. Genes are annotated on the x-axis. Segments without significant BLAST matches are shaded.

To identify the *E* and *I* silencers in the *sensu stricto* species, we searched for peaks of conservation in multiple sequence alignments. For both of the *S. cerevisiae HML* and *HMR*, we identified contigs in the sequenced *sensu stricto* species that contained a part of the locus and the adjacent gene. The right side of *HMR* was misassembled in *S. paradoxus* with two disjointed contigs with incorrect inverted ends, so we resequenced and assembled the region (GenBank EU597267). *HML* and *HMR* were conserved across all five species with clearly conserved orthologs of the neighboring genes (Table S1). However, unlike most intergenic sequences in the genome, the regions around *HML* and *HMR* were too diverged to allow multiple alignments. Moreover, local pairwise alignments of these flanking sequences between any of the ten species pairs were also unexpectedly dissimilar. The best pairwise alignments were between the two closest species *S. cerevisiae* and *S. paradoxus*, but instead of the genome-wide average of 80% identity for orthologous intergenic regions, the percent identities were: 46% left of *HML*, 55% right of *HML*, 52% left of *HMR*, 45% right of *HMR*. These alignments were almost as dissimilar as if the sequences were unrelated; 1000 random equal-length sequences with identical base composition that we generated had an averaged local pairwise similarity of 45%. BLAST-based comparisons also did not reveal matches for the sequences between HML or HMR and the nearest flanking genes, ruling out local inversions and rearrangements (Figure 2).

Translocations or transpositions could, in principle, have lead to poor alignments across the species in the *HML* and *HMR* flanking regions. In such a case, sequence searches from one species would be expected to produce matches in non-syntenic positions of other species. However, BLAST searches with the diverged intergenic segments around *HML* and *HMR* from each of the five species against the assembled genomes of the other species did not produce significant BLAST results outside of the syntenic contigs. The only exceptions were the *S. cerevisiae* to *S. paradoxus* matches in repetitive DNA (Figure S1); however these likely reflect homogenization of these repeated sequences by gene conversion rather than functional conservation [16]–[18]. We also excluded the possibility that systematic misassembly occurred in these regions in the *sensu stricto* by performing BLAST searches against the unassembled traces of each species. Therefore, sequence assembly issues and rearrangements did not explain the poor alignments of DNA sequences flanking *HML* and *HMR*.

### Conservation of Silencer Sequences within Highly Diverged Intergenic DNA

**Figure 3:**
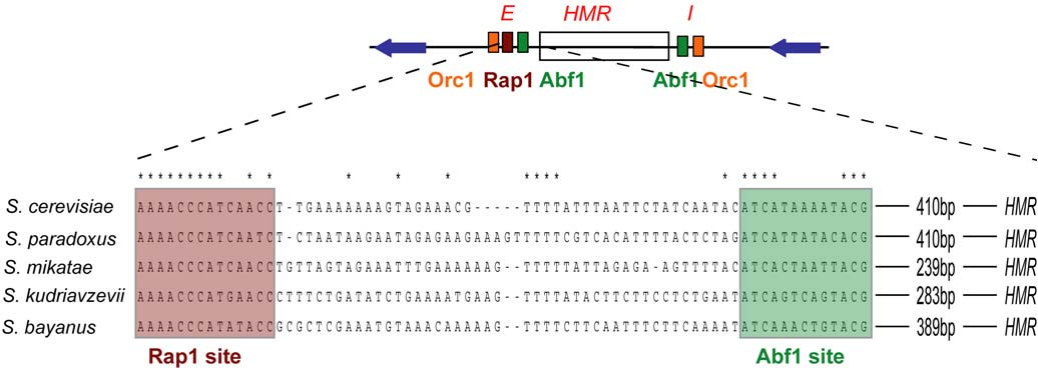
Conservation of the *HMR-E* silencer in five *sensu stricto* species. Multiple alignment of the putative *HMR-E* silencer in the five *sensu stricto* species. There was strong conservation among all of the species of the Rap1 and Abf1 binding sites (shaded) and the spacing between them, with diverged intervening sequence from the Rap1 site to the Abf1 site. Similarly, even though the distance from the Abf1 site to *HMR* was identical in *S. cerevisiae* and *S. paradoxus*, DNA-level pairwise alignment of the region gave only 55% identity (Figure S1).

We determined that the flanking sequences in the five species were indeed orthologous by analyzing conservation of the silencers that have been identified in *S. cerevisiae*. In three of the four cases (*HMR-E*, *HMR-I*, *HML-I*), there was clear conservation of the known functional binding sites in the silencers, despite the low sequence similarity throughout the intergenic regions. To the right of *HML*, an Abf1 binding site was present 319–321 base pairs past the *HMLα1* stop codon in all five species. At *HMR-I*, the sequence of the Rap1 and Abf1 binding sites, their orientation, distance to *HMR*, and spacing between the binding sites were conserved between *S. cerevisiae* and *S. paradoxus*. Similarly, the Abf1 and Rap1 binding sites in *HMR-E* were conserved in all five species, with virtually the same spacing between the sites (39–43 bp), and the distance to *HMR* was identical in *S. paradoxus* and *S. cerevisiae* (Figure 3, Figure S2).

### Functional Conservation of the *HMR-E* Silencer between *S. cerevisiae* and *S. bayanus*

**Figure 4:**
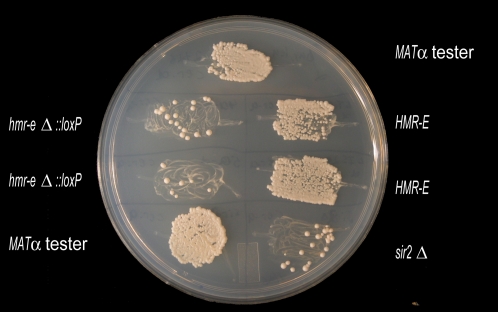
Deletion of the *S. bayanus HMR-E* resulted in loss of silencing. Mating test of *MAT*α strains to *MAT*a tester strain (JRY2726). Disruption of silencing changed the mating type of the *MAT*a strains to nonmating phenotype of a/α diploid. Two independently constructed *S. bayanus hmr-e* deletion strains (JRY8785, JRY8786) lost silencing to the same extent as the *S. bayanus sir2Δ* strain. The parental *HMR-E* strains (JRY8781, JRY8782) mated as efficiently as the *S. cerevisiae* control (JRY2728).

To test if the observed sequence conservation reflected functional conservation, we deleted a 140-bp fragment containing known Abf1p and Rap1p binding sites from the presumptive *HMR-E* in haploid *S. bayanus*. The deletion abolished silencing at the *HMR* locus to the same extent as did deletion of the *SIR2* gene (Figure 4). This experiment, together with the *in silico* observations of the conservation of binding sites and silencer architectures in the *HML* and *HMR* silencers, established that the regions from the five species were orthologous and suggested that the DNA flanking the *HM* loci evolved more rapidly than other intergenic DNA.

### Subtelomeric Intergenic DNA Overrepresented in Highly Diverged Regions

Intrigued by the unusual divergence around *HML* and *HMR*, we sought to determine if other silenced regions were enriched for diverged sequences. We searched all 6,217 *S. cerevisiae* intergenic regions for DNA sequences without significant matches to any of the other *sensu stricto* genomes (Table S2). In subtelomeric regions, defined as the 50 kb internal to each telomere [2], there was an unmistakable enrichment of these non-conserved intergenic sequences. Of the 344 *S. cerevisiae* intergenic regions with no matches to the *sensu stricto*, over 40% were subtelomeric, even though subtelomeric DNA constituted less than 20% of the total analyzed *S. cerevisiae* intergenic DNA (p<10^−10^ by χ^2^-statistic).

In principle, unequal recombination between repetitive elements and transposon activity might have caused sufficient insertions and deletions to result in segments of subtelomeric DNA in *S. cerevisiae* that lacked counterparts in *S. paradoxus*. Therefore we counted intergenic regions with detectable homology but less than 70% identity between the two species (Table S3). If the enrichment of unique sequences in subtelomeric regions were due to insertions and deletions, we would not expect to also see a subtelomeric enrichment of low-identity regions. However, similarly to the excess of unmatched segments, 12% of intergenic subtelomeric DNA had low-identity matches between *S. cerevisiae* and *S. paradoxus*, compared to 7% in the rest of the genome (p<10^−10^ by χ^2^-statistic). Therefore, an excess of insertions and deletions could not be the sole reason for the enrichment of diverged intergenic sequences in subtelomeric regions.

Unmatched and poorly conserved subtelomeric intergenic regions were found on all chromosomes (Table S2, Table S3). Therefore, the higher-than-expected divergence was not unique to *HML*, *HMR*, or the chromosome that bears them, but was a general phenomenon common to silenced regions.

### High SNP Frequency in Sequences Flanking *HML* and *HMR* and in Subtelomeric Intergenic Regions

**Figure 5:**
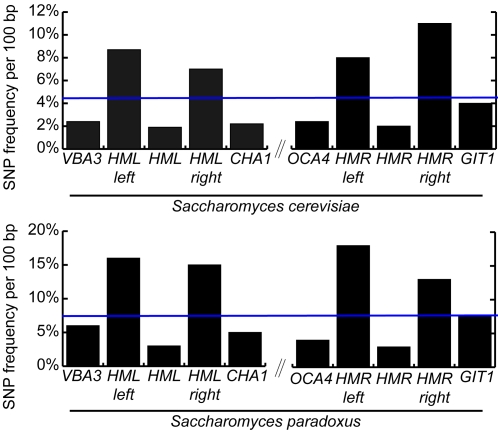
High SNP frequency in *S. paradoxus* and *S. cerevisiae* intergenic regions flanking *HML* and *HMR*. Average percent of SNPs per indicated region in 37 sequenced *S. cerevisiae* and in 27 sequenced *S. paradoxus* strains. The average intergenic SNP frequency in *S. cerevisiae* was 4.5%, and in *S. paradoxus* 7% (blue horizontal lines).

If rapid divergence were an inherent property of silenced DNA, more intra-species polymorphisms in these regions would also be expected. We measured genome-wide average intergenic SNP frequencies in *S. cerevisiae* and *S. paradoxus* [19] and compared them to the frequencies in sequences flanking *HML* and *HMR*. Although the *HML* and *HMR* loci, *per se*, and the four neighboring genes exhibited SNP frequencies typical of genome-wide averages, the intergenic silenced DNA around *HML* or *HMR* had SNP frequencies two to three times higher than average in both species (Figure 5).

**Figure 6:**
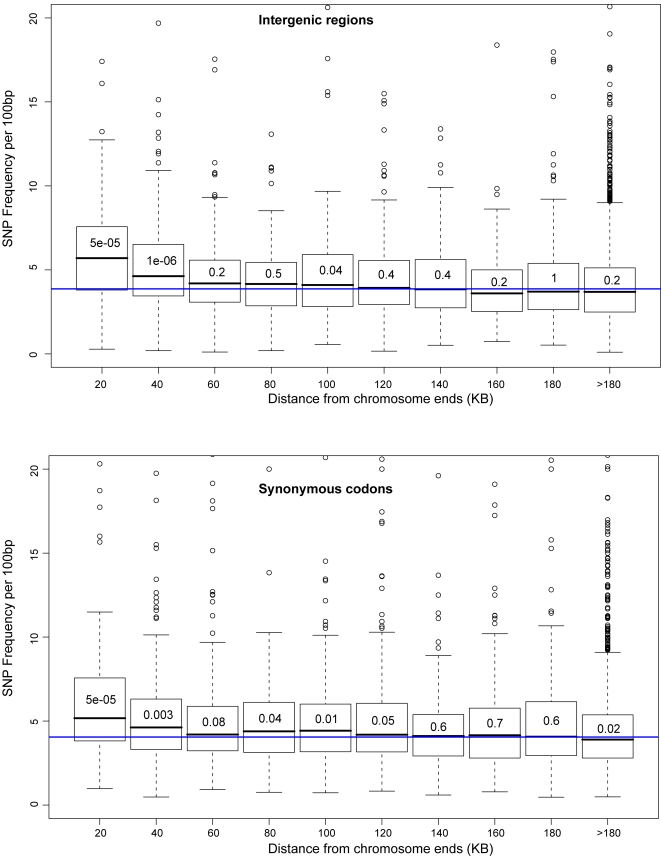
High SNP frequency in subtelomeric *S. cerevisiae* regions. Boxplots of SNP frequencies for intergenic regions and fourfold-degenerate synonymous positions of genes, as a function of distance from telomeres. Only single-copy intergenic and coding regions were included. For codons, only verified genes were considered. Wilcoxon–Mann–Whitney p-values for each distance interval, comparing SNP frequencies against the genome-wide distribution, are indicated within each boxplot.

A similar pattern of SNP frequencies to that observed at the *HM* loci was also detected for telomere-proximal intergenic regions among *S. cerevisiae* isolates. To avoid counting polymorphisms arising from recombination between repetitive DNA sequences, only SNPs in single-copy intergenic regions were considered. SNPs were significantly more frequent in subtelomeric regions, within 0–20 and 20–40 kilobases of telomere edges, than in the rest of the genome (Figure 6, upper panel). The subtelomeric regions were the only ones that deviated strongly from the genome-wide frequencies.

### High SNP Frequency in Synonymous Codons of Subtelomeric Genes

Increased polymorphisms in subtelomeric and *HML* and *HMR*-flanking DNA could result from accelerated base-pair substitutions or from decreased selective constraint on these regions. To distinguish these two possibilities, we analyzed polymorphisms in synonymous positions of codons. If subtelomeric intergenic regions were diverging faster than non-subtelomeric ones because of lower functional constraint, then higher SNP frequencies would be expected for the intergenic but not for synonymous coding positions of subtelomeric DNA.

We counted SNPs at fourfold-degenerate synonymous sites of single-copy genes in *S. cerevisiae*; dubious genes were excluded. Synonymous SNP frequencies in subtelomeric genes were significantly elevated, compared to the rest of the genome, and the level of increase was similar in the synonymous coding and in intergenic positions (Figure 6, lower panel). For the analyzed subtelomeric and non-subtelomeric genes, there was no significant difference in protein-level conservation of orthologs between *S. cerevisiae* and *S. paradoxus* (Wilcoxon–Mann–Whitney p = 0.10) (Figure S3). For the codons of the four genes flanking HML and HMR in *S. cerevisiae*, the fourfold-degenerate synonymous SNP frequency was also elevated compared to the genome-wide average (7% versus 4.4%), however due to the small number of total synonymous sites, the difference was less statistically impressive (p = 0.01 by χ^2^-statistic).

Presumably, fourfold-degenerate synonymous sites of similarly conserved genes are under the same selection, regardless of chromosome position. The concordance between SNP frequencies in intergenic regions and in synonymous codon positions in functional genes implied that the higher SNP frequency closer to chromosome ends resulted from hyperdivergence rather than relaxed selective constraint.

### Transcription-Coupled Repair Did Not Explain Elevated Subtelomeric Substitution

Transcription-coupled repair is a type of the general nucleotide excision repair that targets repair machinery to highly transcribed genes [20]. One possible model is that silenced DNA, by virtue of its lack of expression, is deficient in transcription-coupled repair, resulting in increased substitutions. We tested this possibility by analyzing the effect of expression on SNP frequencies for intergenic and coding regions.

**Figure 7:**
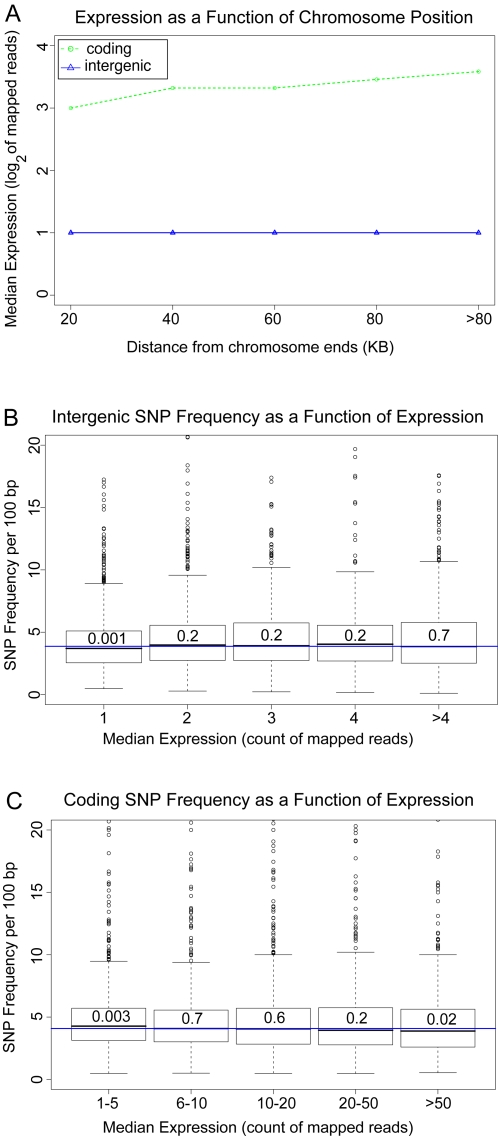
Lack of correlation between expression level and SNP frequency in *S. cerevisiae*. (A) Median expression for intergenic regions and transcripts, as a function of distance from telomeres. (B) Boxplots of SNP frequencies for intergenic regions, as a function of median expression level. (C) Boxplots of SNP frequencies in fourfold-degenerate synonymous positions of genes, as a function of median expression level. Wilcoxon–Mann–Whitney p-values for each expression level, comparing SNP frequencies against the genome-wide distribution, are indicated within each boxplot.

A genome-wide RNA-sequencing dataset [21] was used to assign median expression level for each gene and intergenic region. The extent of expression of intergenic DNA was indistinguishable between the most telomere-proximal and non-subtelomeric regions (Figure 7A). As would be expected from the observation, there was no correlation between intergenic expression and SNP density (Figure 7B). For genes, there was a definite decrease in median expression of subtelomeric genes (Figure 7A). However, as for the intergenic regions, there was no increase in SNP frequencies for highly expressed genes (Figure 7C).

Therefore the lack of coding or non-coding correlation between expression and SNP frequencies indicated that transcription-coupled repair was not likely to have contributed to the hyperdivergence of DNA sequence in silenced regions.

## Discussion

Chromosome ends vary widely among the *sensu stricto* species due to transposons, gene families, and other repetitive elements [2]. By focusing on orthologous sequences that flank the *HML* and *HMR* loci in these species and on unique subtelomeric DNA, we identified an additional contribution to diversification of these regions: increased base-pair substitutions.

The data in this paper were based upon SNP frequencies, which reflect the combined effect of the rate of nucleotide change and repair, and the strength of selection. Because the elevated SNP frequency was also found in silenced regions in synonymous coding positions, the most parsimonious view was that selection had little if any impact on these frequencies. Therefore, we inferred that the increased SNP frequency in silenced chromatin reflected an increased mutation rate; whether that increased rate resulted from increased rates of substitution or repair, or both, could not, at present, be determined.

Our analysis of inter- and intra-species variation detected a clear and compelling correlation between Sir-silenced regions and those that exhibited hyperdivergence. In *S. cerevisiae*, the increase in SNP frequencies was higher in constitutively silenced *HML* and *HMR* regions than in the transiently silenced subtelomeric DNA. We considered a myriad of other explanations including proximity to tRNAs, transposons, LTRs, and autonomous replicating sequences and also base composition; however, none of these genomic features explained the dramatic increase in divergence within subtelomeres and in regions flanking *HML* and *HMR*. Because silencing can interfere with DNA repair, Sir-based silencing appeared to be the most likely mechanism for this rapid sequence diversification. DNA at the expressed *MAT* locus is repaired 2.5 times faster than identical DNA at the silenced *HML* locus [22], and silencing interferes with both photolyase and nucleotide excision repair pathways at a subtelomeric position, independently of transcription [23]. Although Sir-based inhibition of repair was an adequate explanation of these data, we could not exclude the possibility that silenced chromatin may have intrinsically reduced replication fidelity. We considered other possible explanations, of which transcription-coupled repair seemed most plausible, since it should be rendered less useful for genes subject to silencing. However, upon genome-wide analysis, we found no correlation between the level of expression and the frequency of SNPs. Hence, trascription-coupled repair was an unlikely explanation for the increased mutation rate in silenced regions of the genome.

In principle, it should be possible to test whether Sir-based silencing were responsible for the rapid diversification of sequences near and within silenced regions by evolving Sir+ and Sir− strains over a sufficiently long time, and then sequencing the genomes. However, our best estimate of the time that would be required suggested this approach was impractical. There is little doubt that the *URA3* gene, if inserted in silenced regions, could be used to detect a higher frequency of *ura3* mutations in silenced versus non-silenced regions of the genome. However, the phenotypic lag introduced by the higher expression level of *URA3* in the Sir^−^ cells would give the expected correlation of Sir genotype to mutation rate, but for the wrong reason.

Regardless of the underlying mechanism, the potential benefit or detriment to the cell of elevated substitutions in subtelomeres is an intriguing question. Subtelomeric regions are gene poor; therefore the cost of increased mutation rate in these regions might merely be tolerated by the yeasts. However, certain characteristics of heterochromatin in many different organisms and of subtelomeric DNA in yeasts and eukaryotic pathogens raised the possibility that an increased mutation rate may have selective advantage. Heterochromatin in many fungi, animals, and plants commonly contains transposable elements [24],[25]. In budding yeasts, silenced DNA is a hotspot for Ty5 retrotransposon insertion [16], and the Sir4 silencing protein directly interacts with the integrase of Ty5, targeting it to silenced DNA [26]. Silenced chromatin could serve as a decoy to attract an invading transposon to that portion of the genome where its expression would be inhibited, while increased rates of substitution would help to inactivate the newly incorporated transposon [27].

An alternate hypothesis for a beneficial role of hyperdivergence is inhibition of deleterious recombination. Ectopic recombination between repetitive subtelomeric DNA sequences destabilizes the genome. Of the 19 reciprocal translocations identified in the *Saccharomyces* species, 11 are in subtelomeric regions [2]. Subtelomeric sequences may also promote proper segregation of chromosomes by decreasing meiotic recombination in chromosome ends [28]–[30]. Increased divergence and subsequent reduction in sequence identity would be expected to lower both ectopic recombination between subtelomeric repeat elements and meiotic crossovers in chromosome ends.

It is also possible that residence within hyperdivergent regions may facilitate diversity of certain classes of genes. In *S. cerevisiae*, many of the subtelomeric genes play a role in adapting to changes in environmental conditions [31],[32]. Antigenic variation of most eukaryotic pathogenic parasites relies on subtelomerically positioned genes [33]. If silencing-based hypersubstitution also occurs in these pathogens, it may aid in host immune evasion. More broadly, transient subtelomeric silencing combined with accelerated DNA evolution may increase phenotypic diversity, allowing organisms to cope with environmental changes. Of course, increased diversity in perpetually silenced genes would have questionable evolutionary value. However, most subtelomeric genes are only partially silenced, with the level of silencing both variable on a cell-to-cell basis and heritable through multiple cell divisions. The striking exception to hypermutation in heterochromatic genes in our data were the *HML* and *HMR* loci themselves. Because these loci are in frequent recombinational communication with the *MAT* locus, the powerful selection exerted on *MAT* was presumably the force that, through recombination, removed the variation in *HML* and *HMR* that would be expected, based upon our hypothesis.

Two recent studies indicate an elevated substitution rate in X chromosome subtelomeric regions and *Troponin C* gene family members of *Drosophila melanogaster* [34],[35]. Our study established the generality of this effect across taxa, extended it to the full genome analysis, and excluded all proposed mechanisms except for elevated mutation in silenced regions. Given the conservation of heterochromatic hyperdivergence across taxa, it is presumably beneficial and it may be that increased base-pair substitutions contribute simultaneously to genome stability and to adaptive evolution.

## Materials and Methods

### Yeast Strains

All of the yeast strains used in this study are listed in Table 1.

**Table 1:** Yeast strains used in this study.

Strain	Genotype	Source
JRY2726	*S. cerevisiae*, *MAT* **a**, *his4*	
JRY2728	*S. cerevisiae*, *MAT* **α**, *his4*	
JRY7910	*S. paradoxus MAT* **α**, *ho::NatMX*, *ura3-2*	O. Zill
JRY7880	*S. bayanus MAT* **α**, *ho::NatMX*, *his3-1*	O. Zill
JRY7890	*S. bayanus MAT* **a**, *ho::NatMX*, *trp?-1*	O. Zill
JRY8772	*S. bayanus MAT* **α**, *ho::NatMX*, *his3-1*, *ura3Δ::hph*	
JRY8774	*S. bayanus MAT* **α**, *ho::NatMX*, *trp?-1*, *his3-1*, *ura3Δ::hph*	
JRY8775	*S. bayanus MAT* **α**, *ho::NatMX*, *trp?-1*, *his3-1*, *ura3Δ::hph*	
JRY8781	*S. bayanus MAT* **α**, *ho::NatMX*, *trp?-1*, *his3-1*, *ura3Δ::hph*, *hmr-eΔ::loxP-KL_URA3-loxP*	
JRY8782	*S. bayanus MAT* **α**, *ho::NatMX*, *trp?-1*, *his3-1*, *ura3Δ::hph*, *hmr-eΔ::loxP-KL_URA3-loxP*	
JRY8785	*S. bayanus MAT* **α**, *ho::NatMX*, *trp?-1*, *his3-1*, *ura3Δ::hph*, *hmr-eΔ*	
JRY8786	*S. bayanus MAT* **α**, *ho::NatMX*, *trp?-1*, *his3-1*, *ura3Δ::hph*, *hmr-eΔ*	

### *S. Bayanus HMR-E* Manipulation

The *URA3* gene was replaced in *S. bayanus* strain JRY7880 with the *hph* gene (EUROSCARF plasmid pAG32, [36]), producing the *ura3Δ::hph* strain (JRY8772). The resulting strain was crossed to JRY7890 to give JRY8774 and JRY8775 (from two different tetrads). Next, the 138-bp fragment of the putative *S. bayanus HMR-E*, containing matches to the Abf1 and Rap1 binding sites, was deleted through transformation and homologous recombination with a *loxP-K. lactis URA3-loxP* construct (EUROSCARF plasmid pUG72, [37]). In the resulting strains (JRY8781 and JRY8782), the *K. lactis URA3* sequence was excised by expressing the Cre recombinase (EUROSCARF plasmid pSH62, [37]). The *hmr-e* deletion in the final strains (JRY8785 and JRY8786) was confirmed by sequencing. As a result of these manipulations, the original 138-bp putative *HMR-E* sequence was replaced with 134-bp sequence from pUG72, containing one copy of a *loxP* site and flanking nucleotides from the vector (*hmr-eΔ::loxP*).

### Silencing Assay

The phenotypic consequence of the *hmr-e* deletion in *S. bayanus* was assayed by comparing mating ability of the *hmr-eΔ::loxP MAT*α strains (JRY8785 and JRY8786) to the parental *HMR-E* strains (JRY8781, JRY8782). The *S. bayanus* strains were patched onto synthetic dextrose minimal medium plates [38], overlapping patches of *S. cerevisiae Mat* **a** mating tester (JRY2726). Only diploid hybrids resulting from mating would be histidine prototrophs and able to grow. The disruption in *HMR* silencing changed the *MAT*α mating type to the non-mating phenotype of *MAT* **a**/*MAT*α diploids, interfering with the haploid's ability to mate with the *S. cerevisiae Mat* **a** tester.

### Sequencing *S. paradoxus* DNA Flanking the Right Side of *HMR*

*S. paradoxus* genomic DNA was isolated from JRY7910 using the Qiagen Miniprep kit. 5 kb fragment from *HMR* **a1** to *GIT1* was amplified with LongTemplate DNA polymerase PCR (forward primer: CTCCACTTCAAGTTAGAGTTTGGG; reverse primer: TTATTAGCAGTGAGGCGTCAGCCA). 12 primer sets were used in sequencing reactions to produce overlapping fragments along the 5 kb sequence, and the fragments were subsequently manually assembled based on overlap and deposited in GenBank (EU597267).

### Pairwise Alignments

Multiple alignments were made using the ClustalW program [39]. Local pairwise Smith-Waterman alignments [40] between *S. cerevisiae* and *S. paradoxus* sequences flanking *HML* and *HMR* were performed using the EMBOSS “water” program [41] with DNA-matrix, gap-open penalty of 9 and gap-extension penalty of 1. The flanking regions to the left and right of the *HML* and *HMR* loci were based on the annotations in Table S1, using full intergenic regions from the edge of each flanking gene to the nearest *HML/HMR* edge. Estimation of percent identity in local pairwise alignments of unrelated DNA sequence was based on 1000 alignments between 4,000 base-pair, randomly generated DNA sequences with AT content, matching that of the left side of *HMR* (67%).

### BLAST Searches

All BLAST searches were performed using NCBI BLAST [42] without repeat masking (−F F), and with mismatch penalty of −1 (−q −1). For *HML/HMR* BLASTs, e-value cutoff was set at 10^−3^; for all other searches, the cutoff was 10^−5^. The “blastp” program was used for *S. cerevisiae* and *S. paradoxus* orthologous protein comparisons; and the “blastn” program was used for all other intergenic and coding DNA BLASTs.

### Subtelomeric versus Non-Subtelomeric Intergenic Conservation

Intergenic regions of *S. cerevisiae* were defined as sequences between transcript edges of all SGD-annotated genes, including uncharacterized, dubious, and coding regions. Transcript edges were defined using the annotations from the RNA-sequencing dataset [21], to exclude 5′ and 3′ untranslated regions from the intergenic sequence. Overlapping BLAST matches to *S. paradoxus* were merged into contiguous blocks, regardless of synteny. *S. cerevisae* intergenic sequences 250 base-pairs or longer without BLAST results were considered unmatched. In analysis of poorly conserved intergenic DNA, BLAST matches with less than 70% identity were compared to matches with greater than 70% identity.

### SNP Analysis

*S. cerevisiae* and *S. paradoxus* SNP positions were downloaded from http://www.sanger.ac.uk/Teams/Team71/durbin/sgrp [19]. SNPs within 50 kilobases of chromosome ends were counted as subtelomeric, and those at greater distances as non-subtelomeric. Single-copy genes and intergenic DNA were defined as *S. cerevisiae* sequences that produced only a single significant BLAST match to themselves. If any part of an intergenic region or a gene had additional BLAST matches, the whole region or gene was excluded from the SNP analysis. Genes classified as “dubious” in the *Saccharomyces* Genome Database were not considered.

### Expression Analysis

Expression levels were obtained from the genome-wide RNA-sequencing dataset [21]. For each transcript and intergenic region, expression level was defined as the median of all the mapped RNA sequencing reads from that segment. SNP frequencies, as described above for the intergenic and synonymous coding regions, were graphed against the respective expression levels, as indicated on the x-axes of Figure 7B and Figure 7C.

### Ortholog Conservation between *S. cerevisiae* and *S. paradoxus*

*S. paradoxus* orthologs of *S. cerevisiae* genes were determined based on best-reciprocal BLAST matches. All possible peptide sequences longer than 50 residues were extracted from six-frame translation of the *S. paradoxus* genome. Verified and uncharacterized SGD-annotated *S. cerevisiae* proteins were BLASTed against all the potential *S. paradoxus* peptides. For each *S. cerevisiae* protein (X^C^), the best *S. paradoxus* match (X^P^) was then BLASTed back against all *S. cerevisiae* proteins, and if the best match for X^P^ was also X^C^, the pair was defined as orthologous. For the genes used in SNP analysis (non-dubious and single-copy in *S. cerevisiae*), distribution of protein percent identity of subtelomeric *S. cerevisiae—S. paradoxus* orthologs was compared to orthologs positioned greater than 50 kilobases from chromosome ends in *S. cerevisiae*.

### Statistical Analyses

All statistical tests were performed using R [43].

## Supporting Information

Figure S1 Lack of conservation in *HML* and *HMR* flanking intergenic regions. BLAST searches with *S. cerevisiae HML* and *HMR* and surrounding sequence against *S. paradoxus*. Upper panel shows BLAST results against syntenic *S. paradoxus* contigs that contain *HMR* and *HML*. Lower panel displays BLAST results with the same *S. cerevisiae* query sequence against the entire genome of *S. paradoxus*. Percent identity is plotted in 200-bp windows. Genes and mating loci are annotated on the x-axis. Segments without significant BLAST matches are shaded. Additional matches around *HMR* from searches against all of *S. paradoxus* were mostly due to repeated sequences, as can be seen from the stacking of matches (compare upper and lower panels of *HMR*).
(1.55 MB TIF)

Figure S2 *S. cerevisiae* and *S. paradoxus* lack of sequence conservation between *HMR-E* Abf1 binding site and *HMR*. Pairwise global alignment (Needleman-Wunsch) of DNA sequence between the *HMR-E* Abf1 binding site and the *HMR* edge, comparing *S. cerevisiae* to *S. paradoxus*. Length of the intervening sequence between *HMR-E* and *HMR* was identical in both species, but sequence conservation itself was poor.
(0.00 MB TXT)

Figure S3 Similar conservation of subtelomeric and non-subtelomeric genes between *S. cerevisiae* and *S. paradoxus*. Distributions of protein-level percent identities between *S. cerevisiae* and *S. paradoxus* orthologous genes, comparing subtelomeric versus non-subtelomeric genes. No significant difference in cross-species conservation of subtelomeric versus non-subtelomeric orthologs was evident (Wilcoxon-Mann-Whitney p = 0.10).
(1.16 MB TIF)

Table S1 Annotation of the *sensu stricto* contigs corresponding to *S. cerevisiae HML* and *HMR* loci.
(0.01 MB XLS)

Table S2 *S. cerevisiae* intergenic regions with no BLAST matches in *sensu stricto* species.
(0.01 MB TXT)

Table S3 *S. cerevisiae* intergenic regions with less than 70% identity matches in *S. paradoxus*.
(0.02 MB TXT)
