# Supplementary figures and images for: Silent but Not Static: Accelerated Base-Pair Substitution in Silenced Chromatin of Budding Yeasts
Source: PLoS Genet. 2008 Nov 7;4(11):e1000247. doi: 10.1371/journal.pgen.1000247 (PMC2570616; doi:10.1371/journal.pgen.1000247)

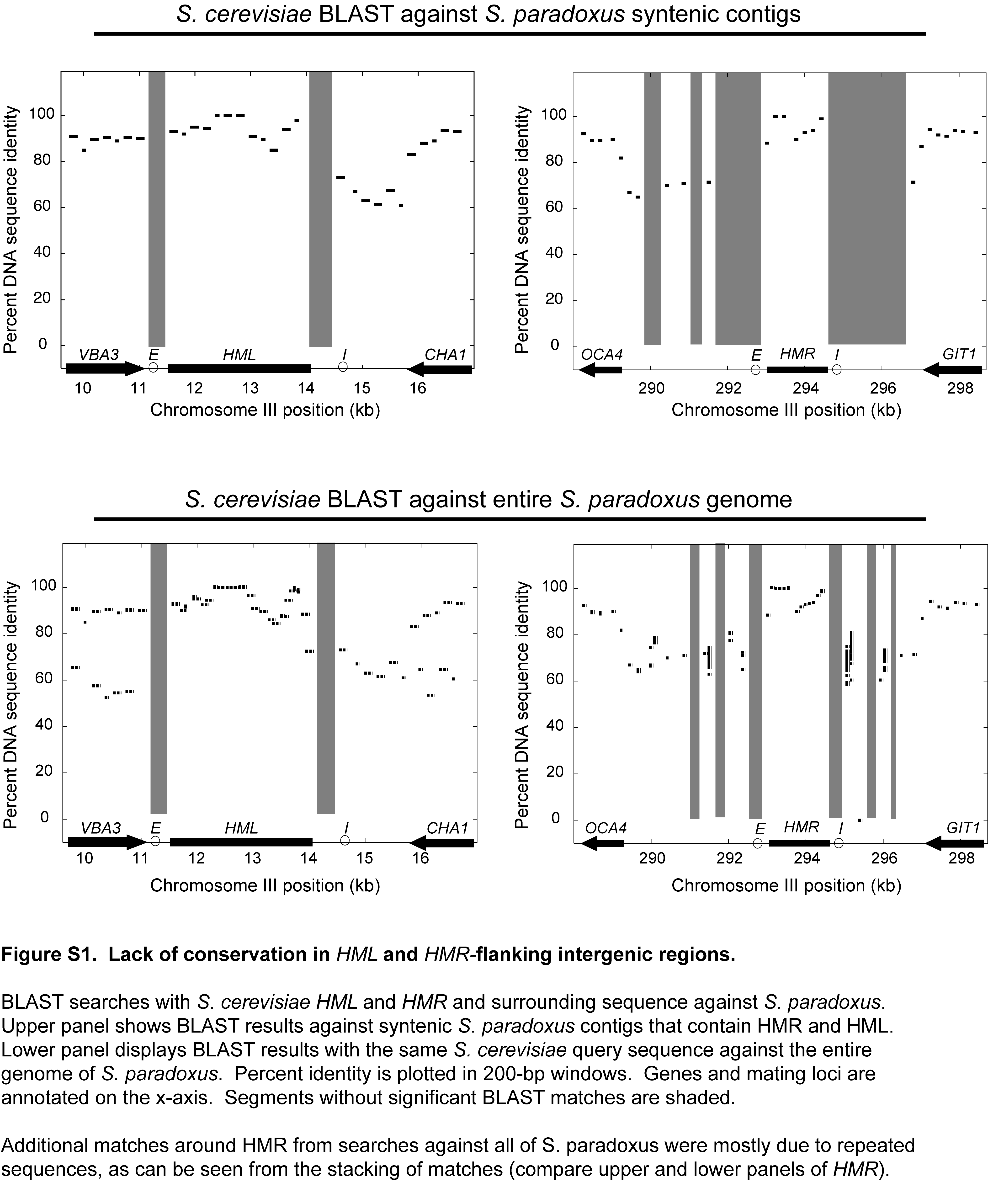

Supplement: Figure S1 — Lack of conservation in HML and HMR flanking intergenic regions. BLAST searches with S. cerevisiae HML and HMR and surrounding sequence against S. paradoxus. Upper panel shows BLAST results against syntenic S. paradoxus contigs that contain HMR and HML. Lower panel displays BLAST results with the same S. cerevisiae query sequence against the entire genome of S. paradoxus. Percent identity is plotted in 200-bp windows. Genes and mating loci are annotated on the x-axis. Segments without significant BLAST matches are shaded. Additional matches around HMR from searches against all of S. paradoxus were mostly due to repeated sequences, as can be seen from the stacking of matches (compare upper and lower panels of HMR). (1.55 MB TIF) [file pgen.1000247.s001.tif]

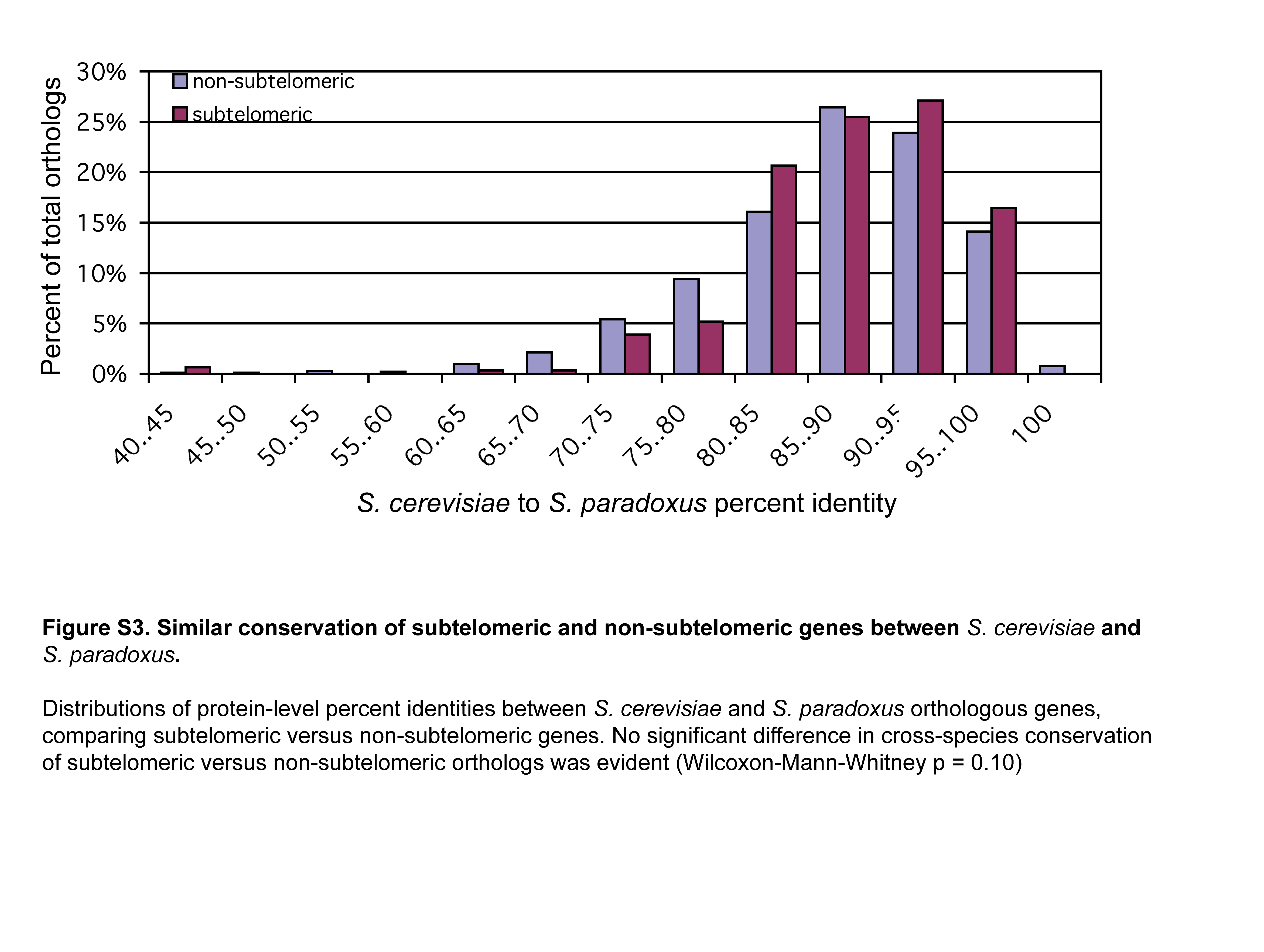

Supplement: Figure S3 — Similar conservation of subtelomeric and non-subtelomeric genes between S. cerevisiae and S. paradoxus. Distributions of protein-level percent identities between S. cerevisiae and S. paradoxus orthologous genes, comparing subtelomeric versus non-subtelomeric genes. No significant difference in cross-species conservation of subtelomeric versus non-subtelomeric orthologs was evident (Wilcoxon-Mann-Whitney p = 0.10). (1.16 MB TIF) [file pgen.1000247.s003.tif]
